# Supplementary material for: Targeting p53 and histone methyltransferases restores exhausted CD8+ T cells in HCV infection
Source: Nat Commun. 2020 Jan 30;11:604. doi: 10.1038/s41467-019-14137-7 (PMC6992697; doi:10.1038/s41467-019-14137-7)
Supplement: Supplementary file 1 — Supplementary Information [file 41467_2019_14137_MOESM1_ESM.pdf]

Supplementary Information:

Targeting p53 and histone methyltransferases restores exhausted CD8+ T cells in HCV infection

Barili et al.

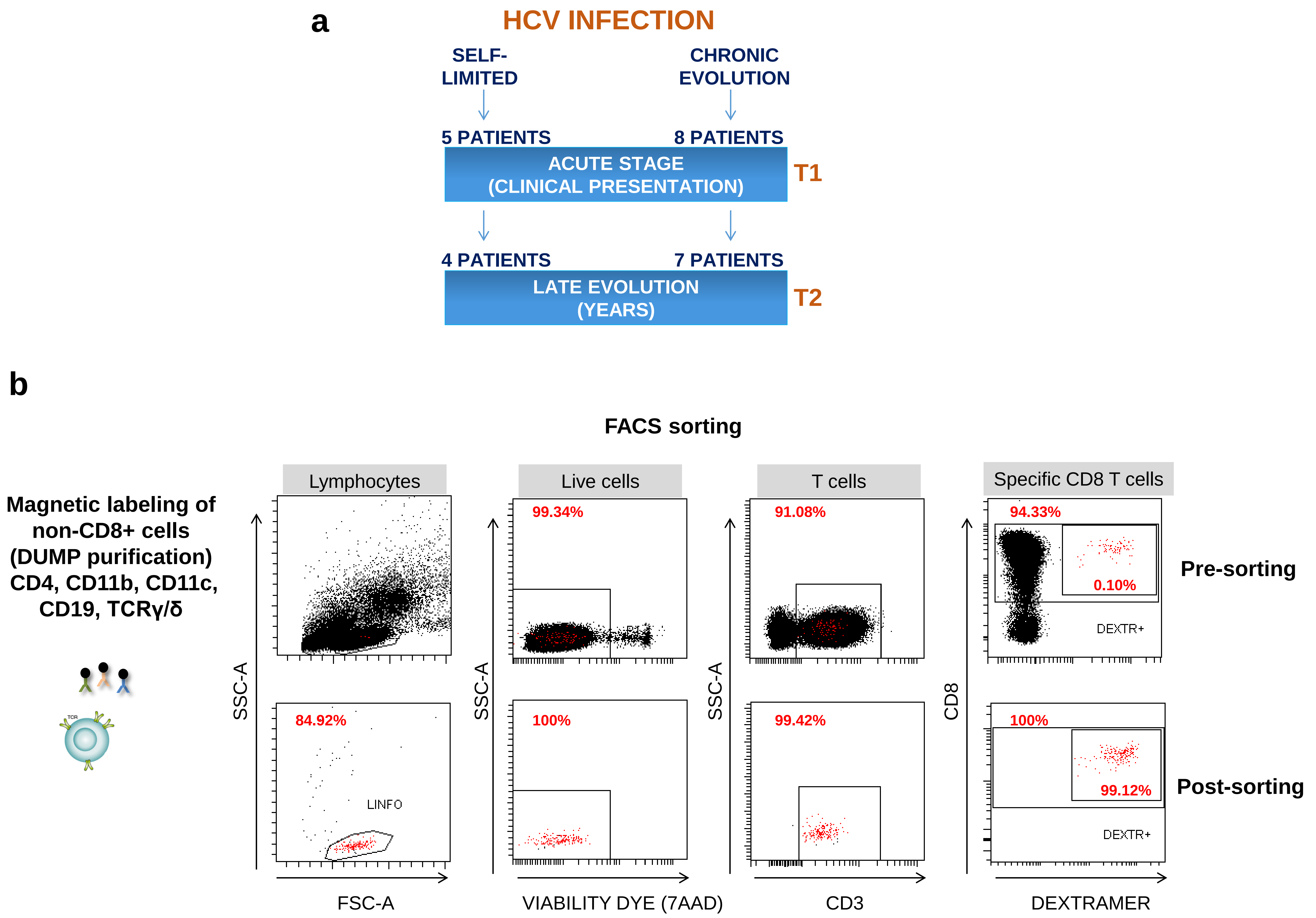

Supplementary Figure 1. Experimental design for the transcriptional profiling of HCV-specific CD8+ T cells

Panel **a** shows the number of patients studied at each time point after infection. Panel **b** illustrates representative flow-cytometry profiles of HCV-specific CD8+ T cells. *Top*, pre-sorting frequency of HCV dextramer-positive cells within the overall CD8+ T cell population after CD8+ T cell enrichment with magnetic beads. *Bottom*, post-sorting purity level of sorted dextramer-positive CD8+ T cells. Frequency of live cells by 7AAD staining and frequency of CD3+ T cells is also illustrated in the *middle* panels.

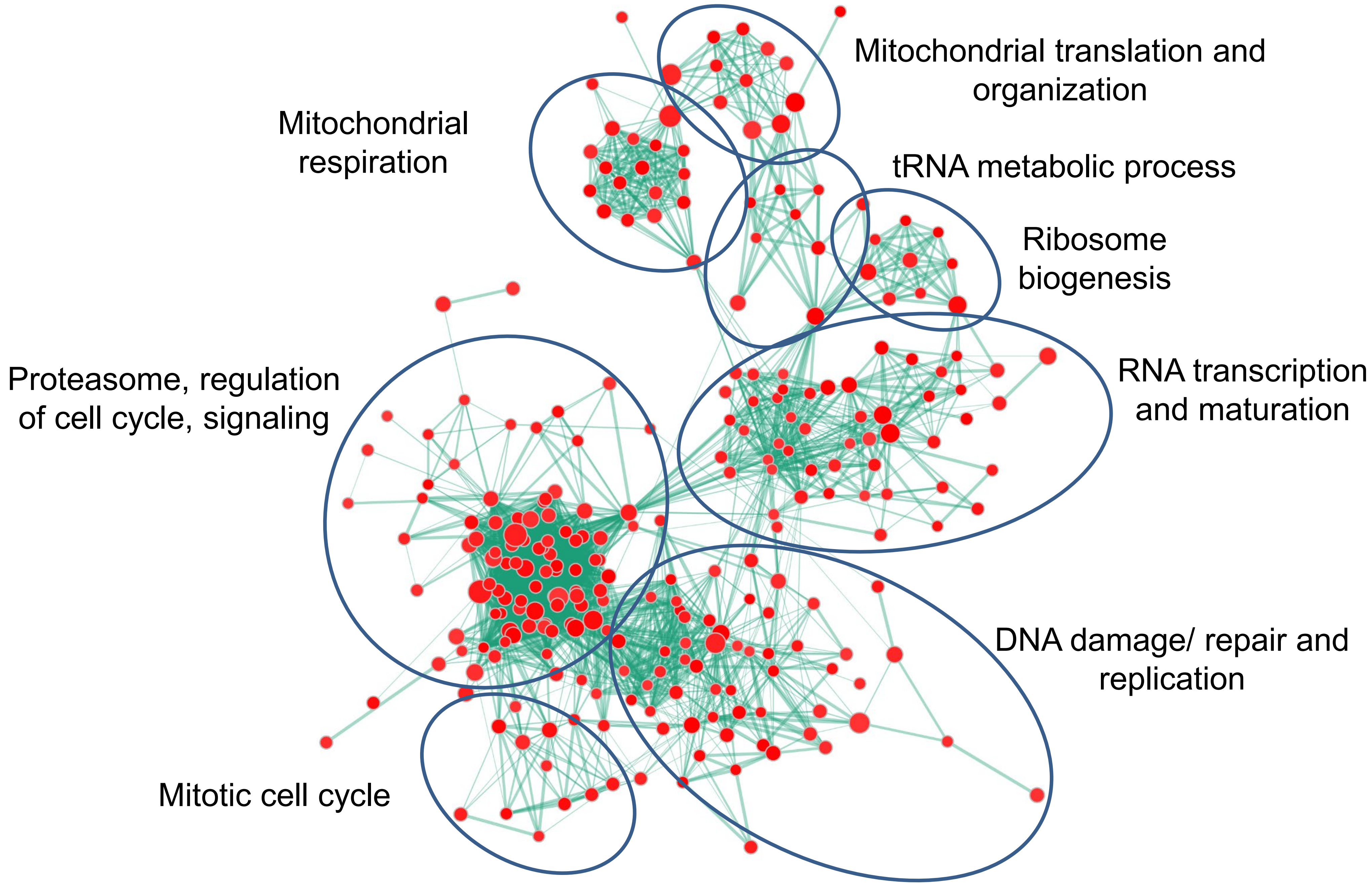

Supplementary Fig. 2. GSEA analysis of gene-expression profiles in HCV-specific CD8+ T cells from acute patients. Network analysis (Cytoscape enrichment map) of significant gene sets from dysregulated pathways (C2 collections) and gene ontology terms (C5 collections) revealed by GSEA at T1/early in chronically evolving as compared to self-limited acute patients. A complete gene set list is given in Supplementary Data 2. Only significantly enriched terms were considered (FDR≤0.1). The node color is dependent on normalized enrichment score (NES) values (red, positive value, up-regulated gene set; blue, negative value, down-regulated gene set). Only red nodes are depicted because down-regulated nodes were not significant; the node size is proportional to the number of genes present in the gene set and the line thickness is proportional to the overlap between gene sets.

T1/early chronically-evolving vs. self-limited acute patients

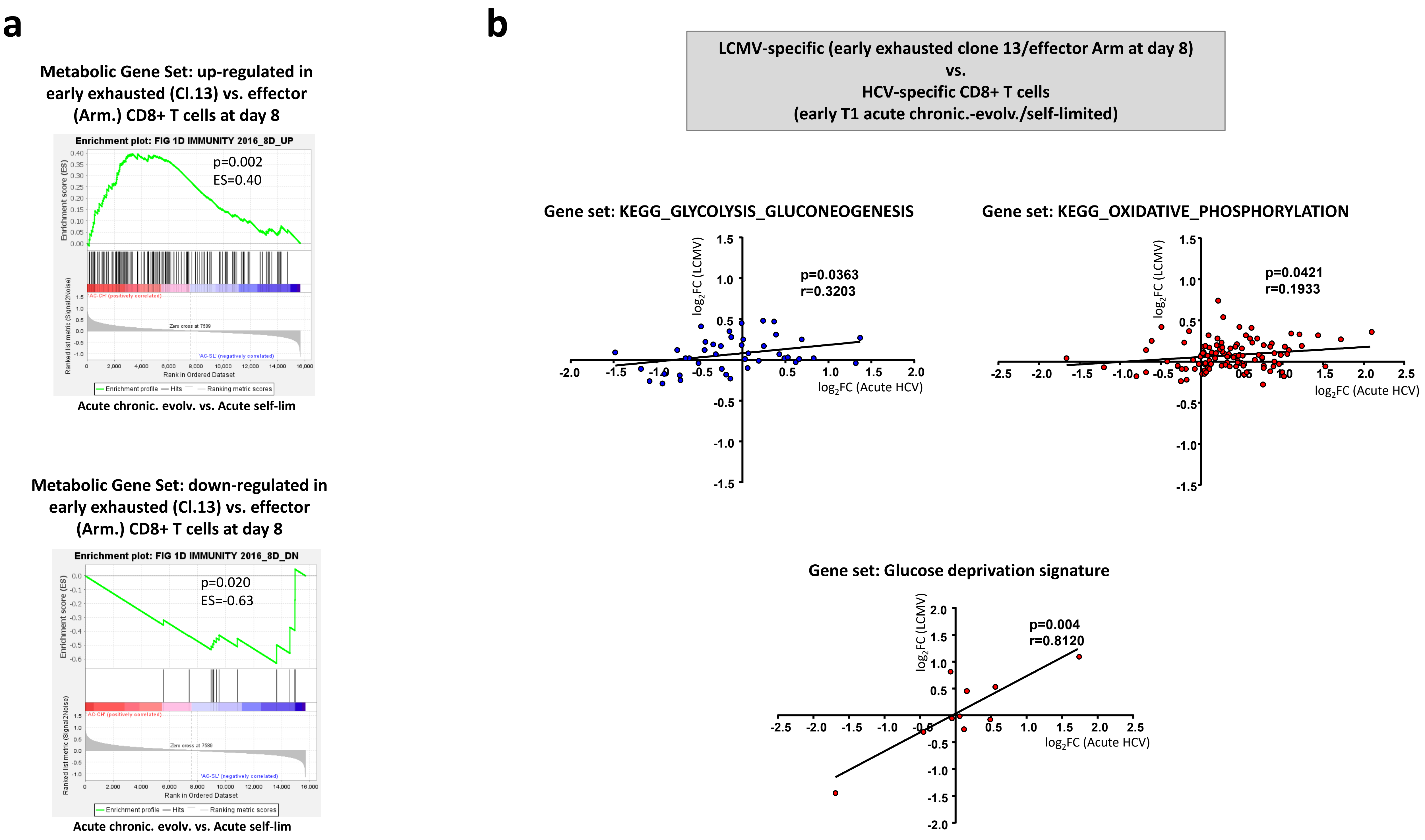

Supplementary Fig. 3. Concordant dysregulation of metabolism-related genes in early LCMV- and HCV-specific exhausted CD8+ T cells.
a. Concordant enrichment of both up- and down-regulated metabolism-related genes (identified in Bengsch et al.)[25] in LCMV clone 13-specific vs. LCMV Armstrong strain-specific CD8+ T cells (day 8 post-infection)[25] with the transcriptome profiling of HCV-specific CD8+ T cells from chronically-evolving vs. self-limited acute patients. **b.** Correlation analysis (Pearson correlation coefficient) of the expression levels of genes comprised within energy-related KEGG pathways (glycolysis and mitochondrial oxidative phosphorylation) and the glucose deprivation signature (as defined by Ho et al., 2015)[29] derived from transcriptome analysis of mouse LCMV infection (early exhausted LCMV clone 13-specific vs. LCMV Armstrong strain-specific CD8+ T cells at day 8 post-infection; data from Doering et al, 2012, publicly available via GSE41867)[68] and human HCV infection (T1/early HCV-specific CD8+ T cells from acute chronically-evolving vs. self-limited patients). In *blue*, Kegg-metabolic pathway enriched in downregulated genes and in *red,* those enriched in upregulated genes.

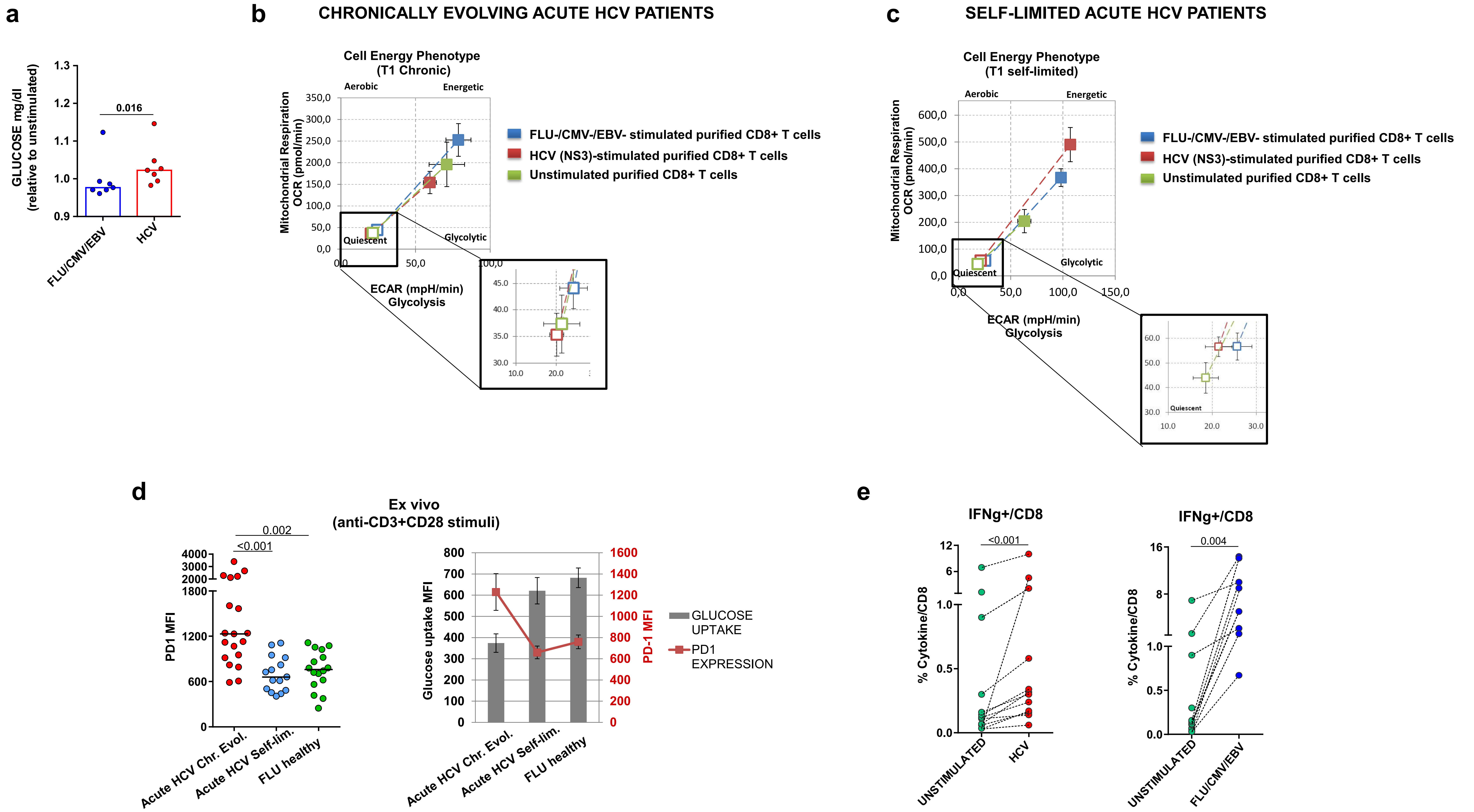

Supplementary Fig. 4. Metabolic dysfunction and PD-1 expression in HCV-specific CD8+ T cells from chronically evolving patients and self-limited acute patients.
a. Glucose consumption was measured in culture supernatants of purified CD8+ T cells from chronically-evolving acute patients, stimulated overnight with HCV-NS3 peptides (red dots) and with control FLU, CMV and EBV peptides (blue dots). Data are presented as the ratio between stimulated and unstimulated cells. Columns represent the median value. Statistical analysis was done with the two-tailed Wilcoxon-matched-paired test (6 patients). Basal and oligomycin stressed ECAR, from metabolic flux profiling of purified CD8+ T cells stimulated as in Fig.**2d** and **2e**, were plotted against basal and FCCP stressed oxygen consumption rates (OCR) to generate the energy phenotype characterization of virus-specific CD8+ T cells from T1/early chronically-evolving (n=6) and self-limited (n=4) acute patients (**b** and **c**, respectively). Data are presented as mean ECAR/OCR ± SEM (n= 6 and 4 patients, respectively). **d.** PD-1 expression (MFI) was measured on virus-specific CD8+ T cells from patients in the acute phase of HCV infection (34 patients) or healthy controls (n=16) after overnight PBMC anti-CD3/anti-CD28 stimulation (*ex vivo* staining). On the *right*, glucose uptake (MFI, median fluorescence intensity) was plotted against PD-1 expression (MFI, median fluorescence intensity) in virus-specific CD8+ T cells. Grey bars and red square symbols represent median glucose uptake and median PD-1 expression ± SEM, respectively. Differences between multiple groups have been evaluated by Kruskal-Wallis non-parametric test. *P*-values have been corrected for pair-wise multiple comparisons, according to the Dunn's method. **e,** IFN-γ production by CD8+ T cells cultured as in **a** from chronically and self-limited acute patients (T1), assessed as intracellular cytokine staining upon HCV or FLU/CMV/EBV peptide stimulation (12 patients for HCV stimuli and 9 for control peptides). Statistical analysis was done by the two-tailed Wilcoxon-matched-paired test.

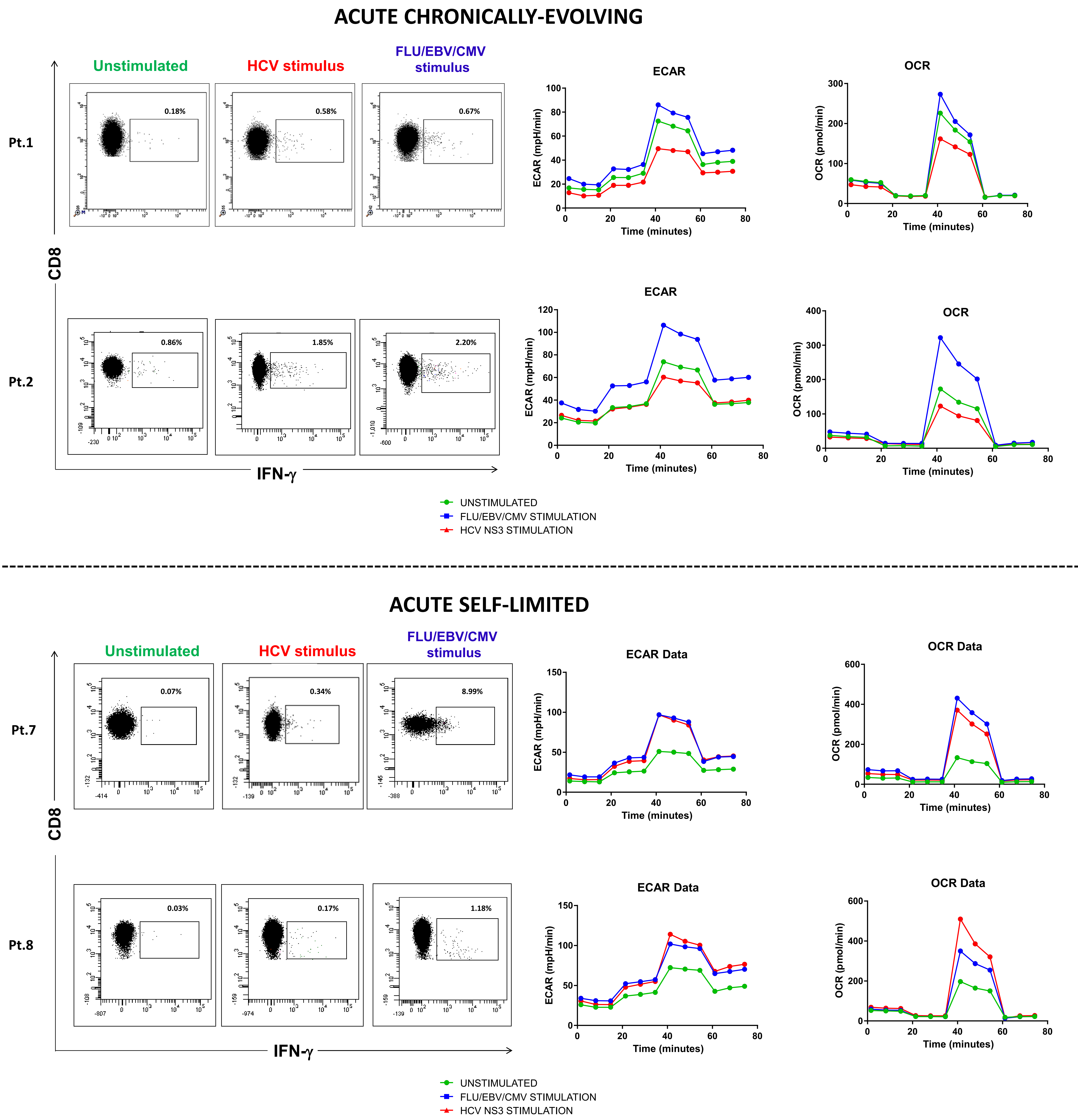

Supplementary Fig. 5. Functional and metabolic profiles of antigen-specific CD8+ T cells of different viral specificities. Representative examples of IFN-γ production (detected by ICS in flow cytometry, *left plots*) and metabolic flux Seahorse profiling (with representation of both ECAR and OCR) of CD8+ T cells either unstimulated (in *green*) or stimulated overnight with HCV-specific peptides (spanning the complete NS3 sequence, in *red*) or control peptides (FLU/CMV/EBV specificities, in *blue*) from chronically-evolving (patients 1 and 2) and from self-limited (patients 7 and 8) acute HCV patients as in Fig. **2d**,**e**, **3d** and **e**.

MIX FLU/CMV/EBV STIMULUS IN T1/EARLY ACUTE CHRONICALLY-EVOLVING

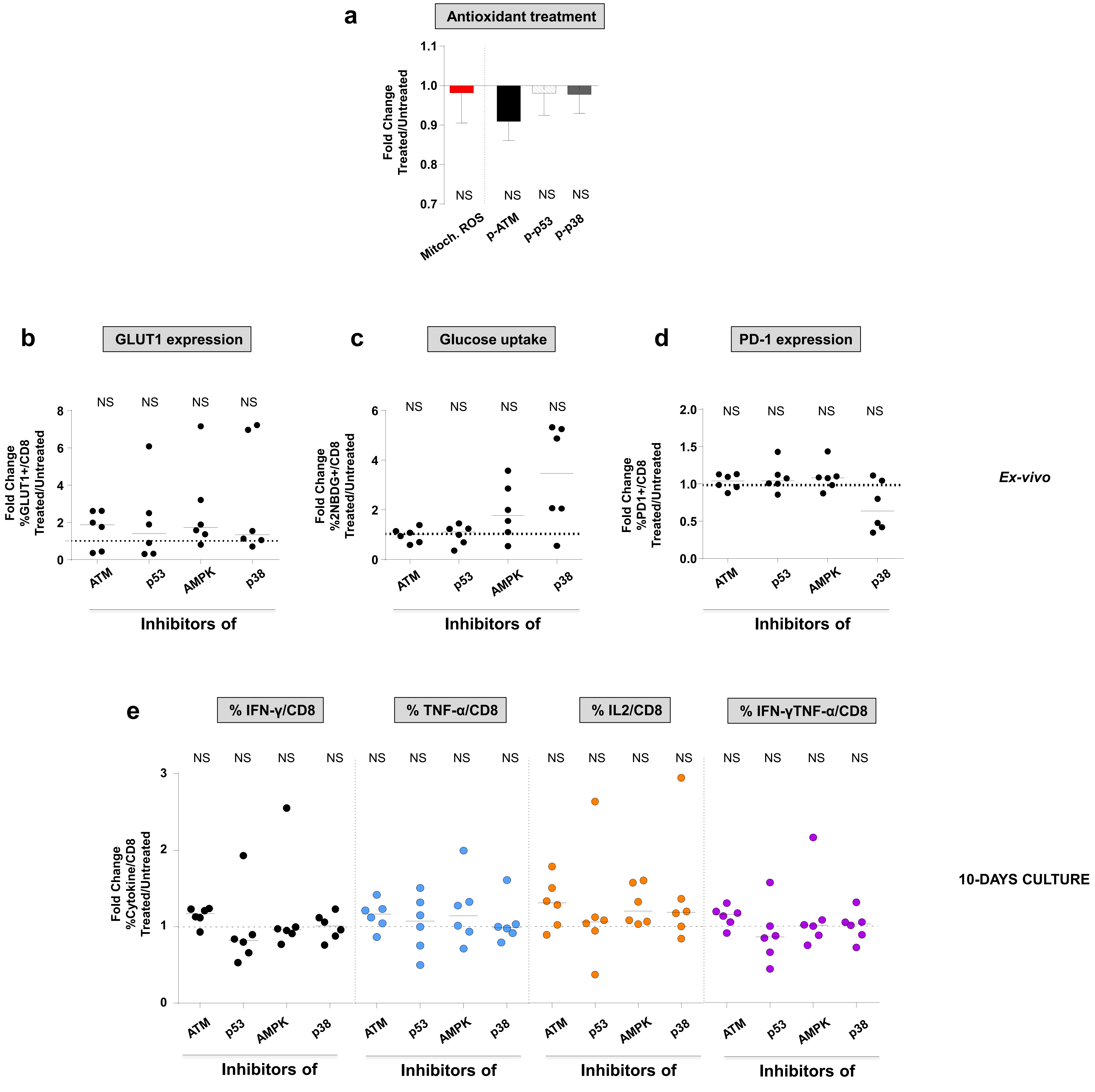

Supplementary Fig. 6. Effect of antioxidant and transducer inhibitors on metabolic functions and cytokine production in chronically-evolving acute patients after viral control peptide stimulation.

a. PBMCs from T1/early chronically-evolving patients (n=6) were stimulated overnight with FLU-/EBV-/CMV-specific peptides in the presence or absence of the ROS scavenger resveratrol, cultured as in **Fig. 5a** and then stained with MitoSOX Red to assess mitochondrial superoxide content and with anti-phospho-ATM (Ser1981), phospho-p38 (Thr180) and phospho-p53 (Ser15). Data are mean values $\pm$ SEM from 6 patients. **b.** to **d.** PBMC from T1/early chronically-evolving patients were stimulated for 40h with FLU-/EBV-/CMV-specific peptides in the presence or absence of specific ATM (KU-55933), p53 (Pifithrin-α), AMPK (Dorsomorphin) and p38a (SB203580) inhibitors, followed by flow cytometry determination of GLUT-1 levels (**b**), glucose uptake measured via incorporation of the glucose analog 2-NBDG (**c**) and PD-1 expression (**d**). Data are presented as fold increase of treated vs. untreated CD8+ T cells (fold-increase). **e,** IFN-γ, TNF-α, IL2 single positive as well as double-positive IFN-γ+/TNF-α+ CD8+ T cells generated in T cell lines upon 10-days stimulation with FLU-/EBV-/CMV-specific peptides in the presence or absence of the inhibitors specified in the legend to panel **b**. Data are presented as the ratio between the percentage of cytokine positive CD8+ T cells detected in inhibitor-treated vs untreated cultures (fold change). Horizontal lines in panels **b** to **e** represent median values; data were statistically evaluated with the Wilcoxon signed-rank test, NS= not significant.

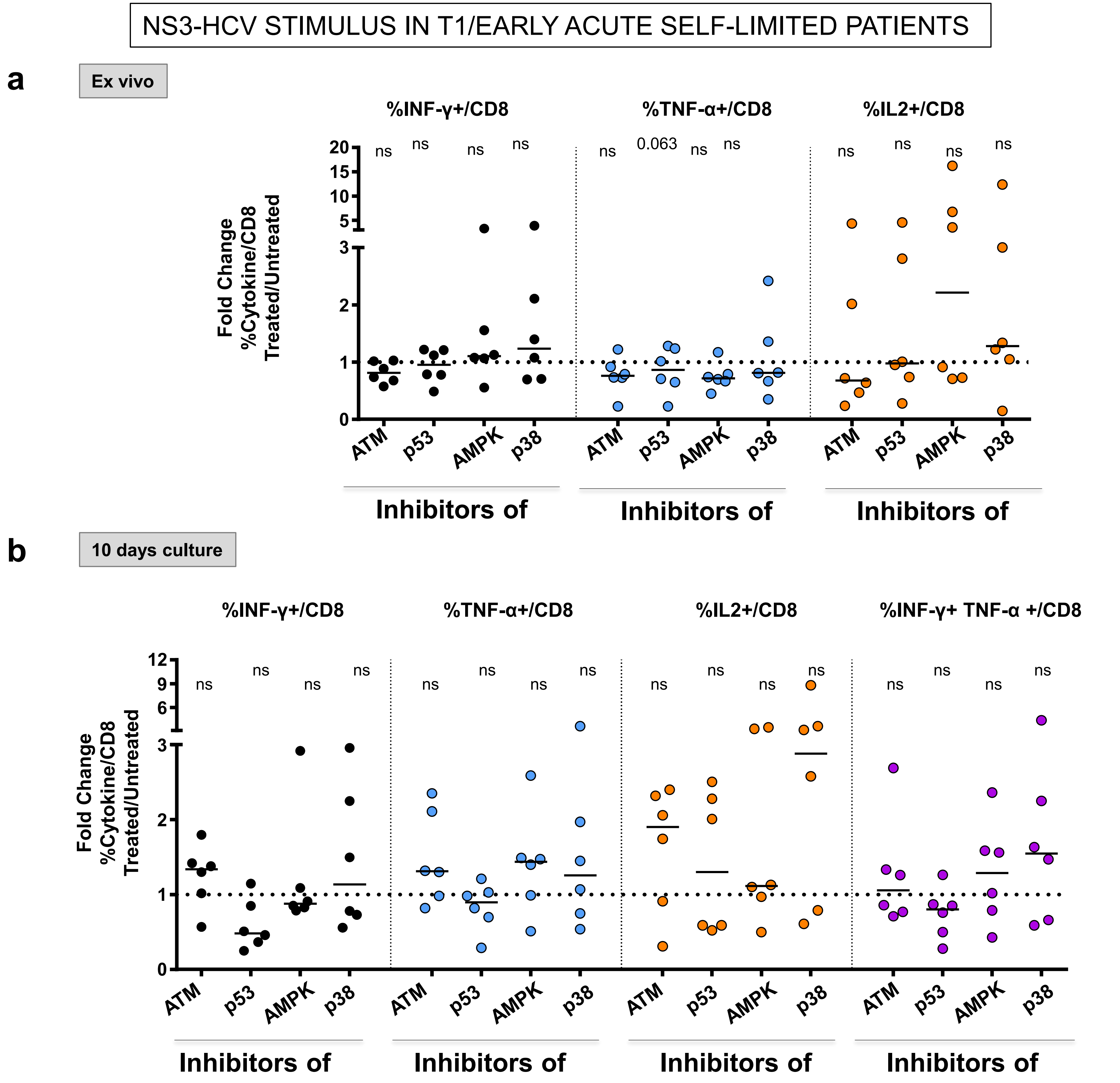

Supplementary Fig. 7. Effect of transducer inhibitors on cytokine production in self-limited acute patients.

PBMCs from T1/early acute self-limited patients (n=6) were stimulated for 40h (**a**) and 10 days (**b**) with HCV-NS3 peptides in the presence or absence of specific ATM (KU-55933), p53 (Pifithrin-a), AMPK (Dorsomorphin) and p38a (SB203580) inhibitors, followed by flow cytometry determination of IFN-γ, TNF-α and IL2 production by CD8+ T cells. Data are presented as the ratio between the percentage of cytokine positive CD8+ T cells detected in inhibitor-treated vs untreated cultures (fold change). Horizontal lines represent median values; data were statistically evaluated with the Wilcoxon signed-rank test, NS= not significant.

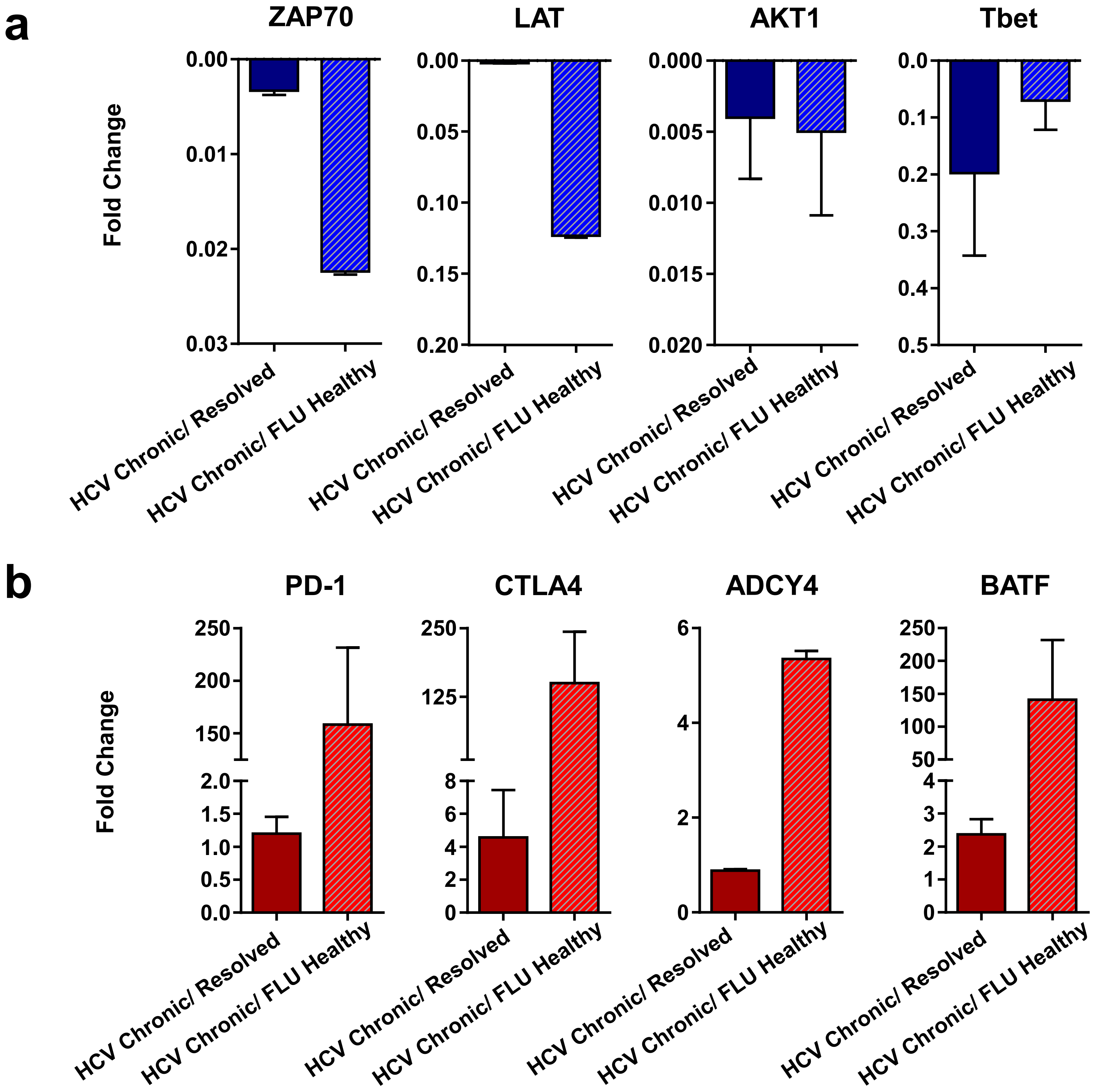

Supplementary Fig. 8. qPCR validation of selected genes associated to T cell exhaustion.
a. Expression levels of TCR downstream effector genes ZAP70 (Zeta-Chain TCR Associated Protein Kinase 70kDa), LAT (Linker for activation of T-cells), AKT1 (AKT Serine/Threonine Kinase 1) and T-Bet (T cell effector-related transcription factor) in HCV-specific CD8+ T cells from chronic *vs.* resolved HCV infection (T2/late) and HCV chronic (T2/late) *vs.* healthy controls. **b.** Expression levels of the exhaustion-related inhibitory co-receptors PD-1 and CTLA-4, the TCR signaling inhibitor ADCY4 (Adenylate Cyclase 4) and the exhaustion-related inhibitory transcription factor BATF (Basic leucine zipper ATF-like transcription factor). Gene expression levels are illustrated as fold change relative to GAPHD expression. In *blue*, fold changes <1 and in *red,* fold changes >1. Data derive from 9 patients (3 for each category). Bars represent mean fold changes ± SEM.

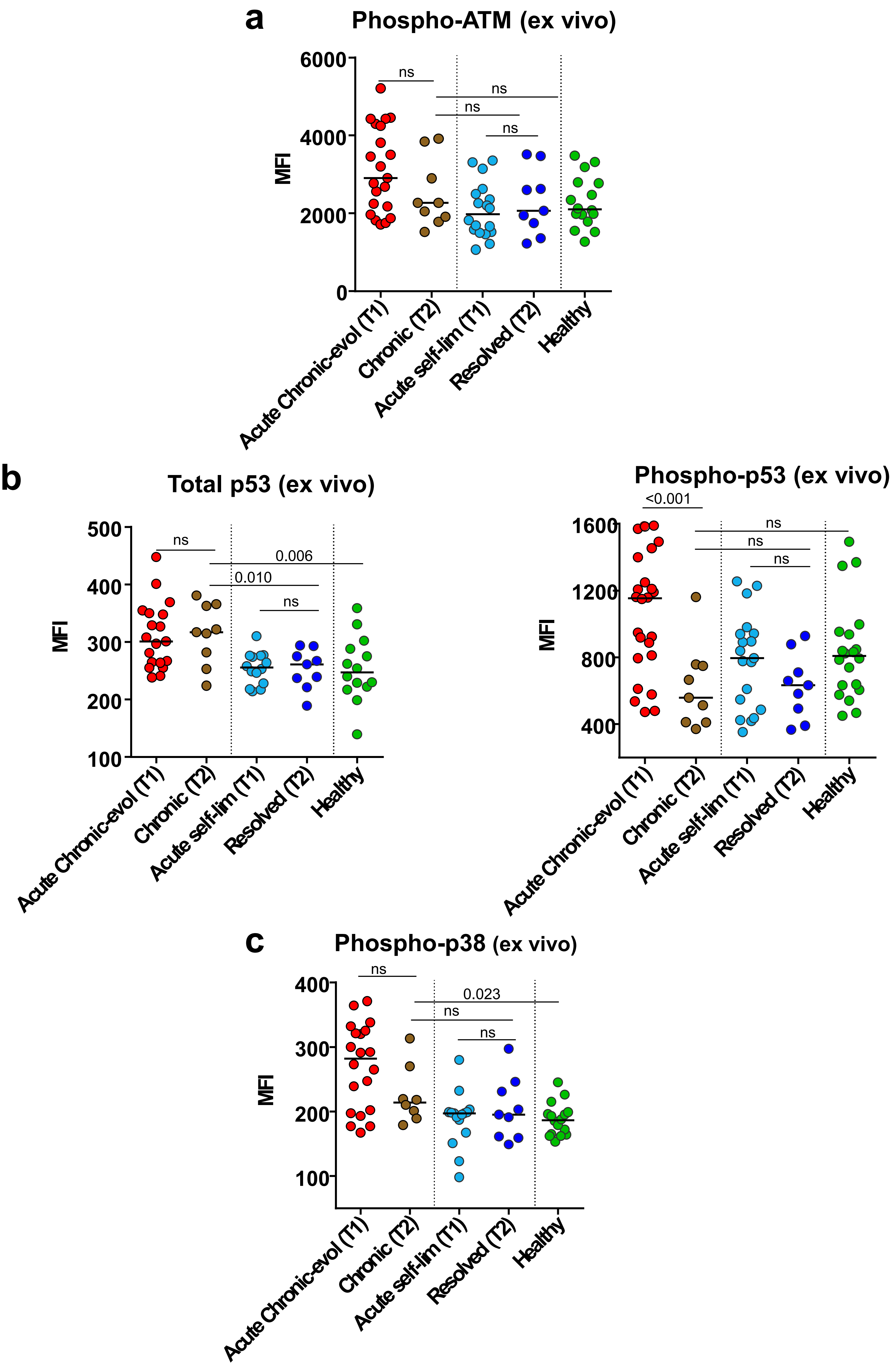

Supplementary Fig. 9. ATM, p53 and p38 intracellular signaling pathways at early and late time points of HCV infection.

a, Intracellular phospho-ATM (Ser1981) staining of dextramer positive virus-specific CD8+ T cells from PBMCs of chronic T1/early and T2/late, self-limited T1/early and T2/late patients, or from healthy controls was performed after overnight stimulation with anti-CD3/anti-CD28 *(ex vivo)*. **b**, Intracellular staining for total p53 and phospho-p53 (Ser15) as in **a**. **c**, Intracellular staining for phospho-p38 (Thr180) as in **a**. Horizontal lines indicate median values from individual patients (shown as single dots). MFI represents median fluorescence intensity. Differences between multiple groups have been evaluated by Kruskal-Wallis nonparametric test. *P*-values have been corrected for pair-wise multiple comparisons, according to the Dunn's method.

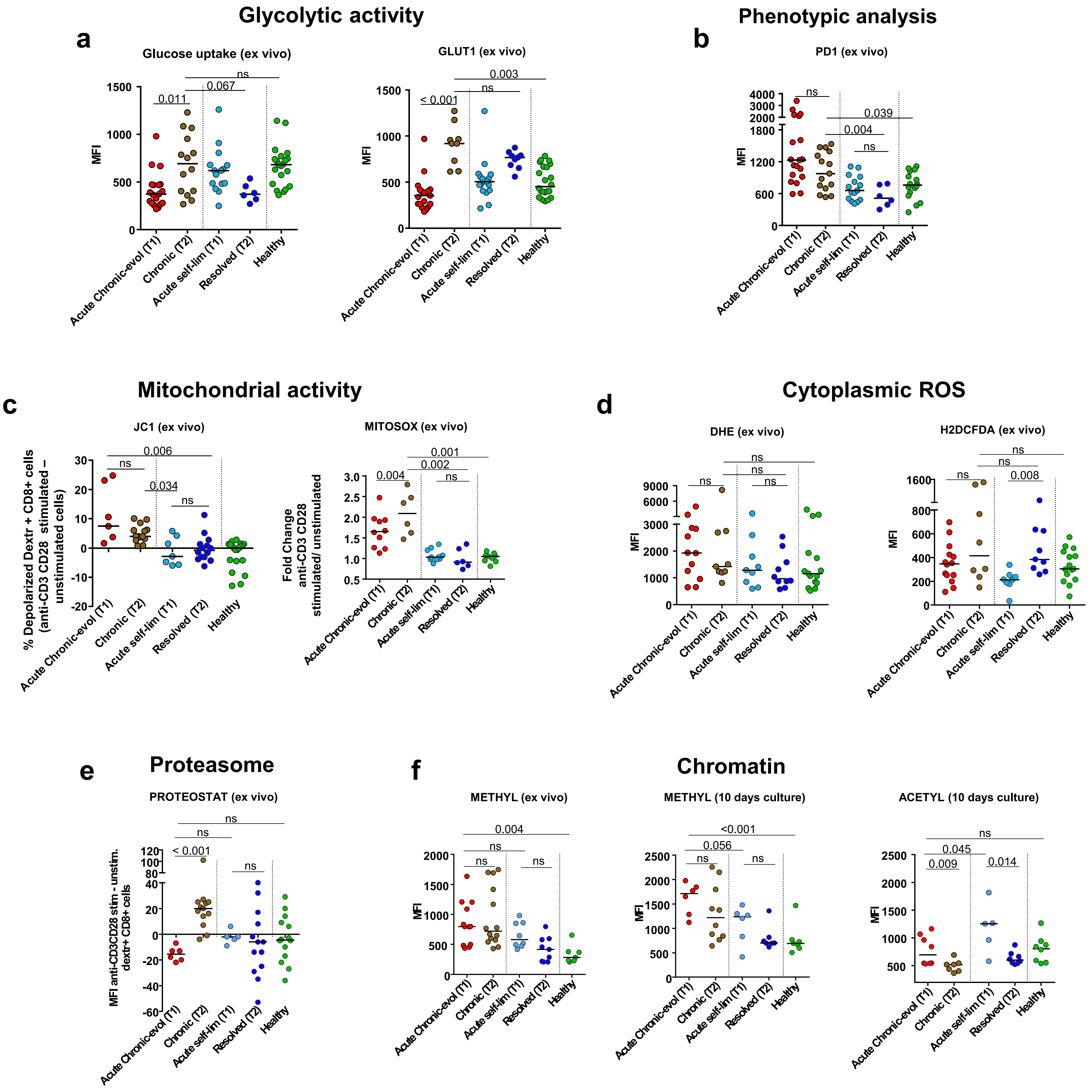

Supplementary Fig. 10. Longitudinal analysis of metabolic, epigenetic and phenotypic parameters in chronic and self-limited patients.
a. Glucose uptake, measured by the incorporation of the glucose analog 2-NBDG (MFI) and Glut1 expression (MFI) in virus-specific dextramer-stained CD8+ T cells from T1/early and T2/late chronic vs T1/early and T2/late self-limited HCV patients and healthy controls after overnight stimulation with anti-CD3/anti-CD28 (*ex vivo*). **b**, PD-1 expression assessed as in **a**. **c**. Percentage of mitochondrial depolarized virus-specific CD8+ T cells determined *ex vivo* (as specified in **a**) by staining with the mitochondrial membrane potential sensitive dye JC-1 (see 'Methods' for details). Dextramer-positive virus-specific depolarized cells were quantified by subtracting the percentage of FL1high/FL2low cells (JC-1 staining) detected in the unstimulated samples from the percentage of the corresponding cellular subsets detected in the stimulated samples, as previously reported[23]. On the right, mitochondrial superoxide levels determined *ex vivo* with the MitoSOX Red dye. **d**, Cytoplasmic reactive oxygen species (ROS) determined *ex vivo* after overnight anti-CD3/anti-CD28 stimulation with the superoxide-specific dye DHE and the intracellular H2O2 specific dye H2DCFDA, as in **a**. **e**, Proteasomal function was measured with the ProteoStat probe (able to bind unfolded protein aggregates) in dextramer-stained HCV-specific CD8+ T cells from chronic and self-limited HCV infection (T1/early and T2/late) and in healthy controls following PBMCs overnight stimulation with anti-CD3/CD28. **f**, Repressive H3K9me2 (left and middle panels) and permissive H3K9ac2 (on the right) histone marks assessed by flow cytometry in dextramer-stained HCV specific CD8+ T cells from chronic and resolved HCV patients (T1/early and T2/late) or healthy controls. PBMCs were stimulated overnight with anti-CD3/CD28 (*ex vivo*) or for 10 days with HLA-A2-restricted HCV- or FLU-peptides. Data are presented as median fluorescence intensity (MFI) values from individual patients (shown as single dots). Horizontal lines represent median values. Differences between multiple groups have been evaluated by Kruskal-Wallis nonparametric test. P values have been corrected for pair-wise multiple comparisons, according to the Dunn's method.

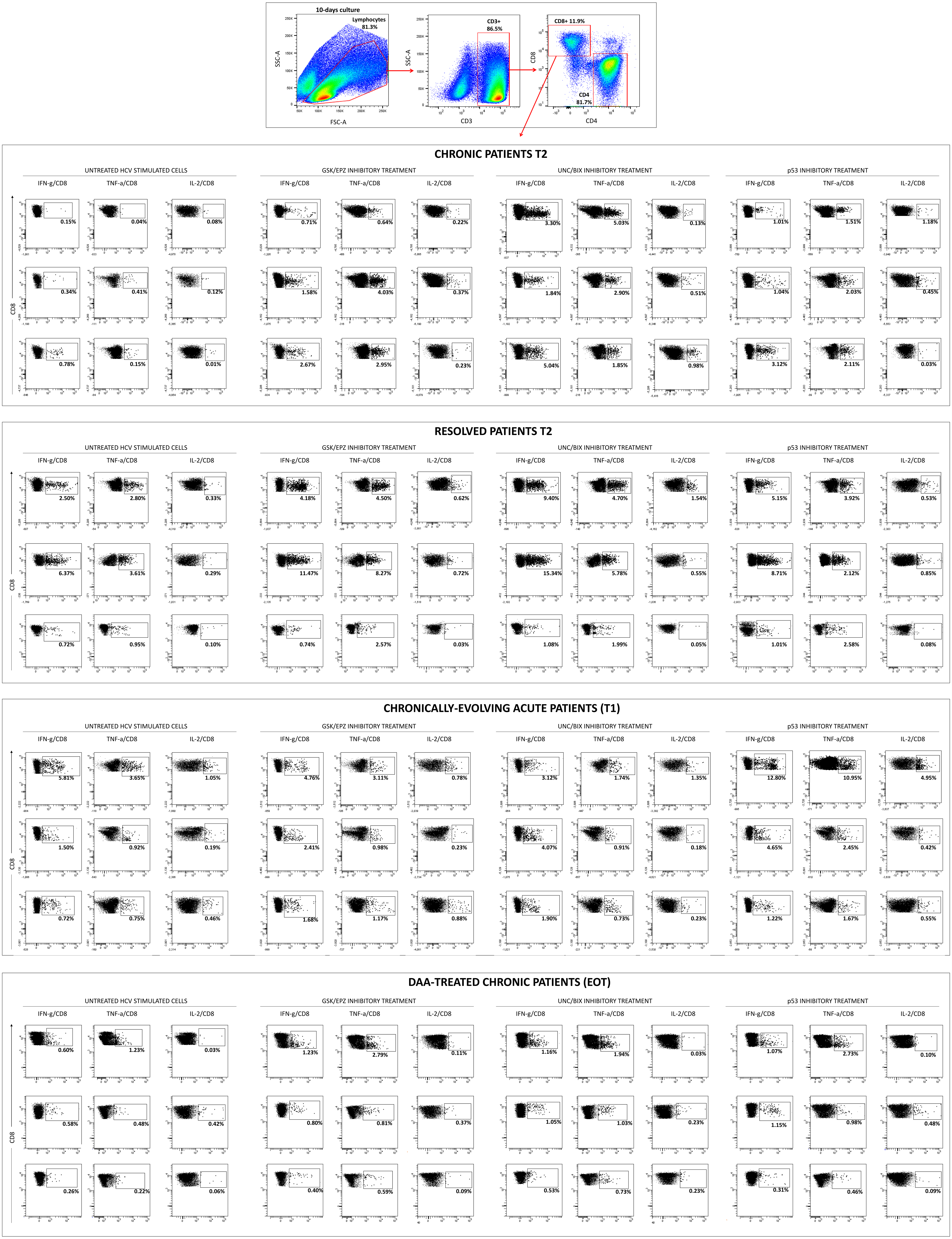

10-days culture
Lymphocytes 81.3%
SSC-A
FSC-A
CD3+ 86.5%
CD3
CD8+ 11.9%
CD8
CD4 81.7%
CD4
CHRONIC PATIENTS T2
UNTREATED HCV STIMULATED CELLS
GSK/EPZ INHIBITORY TREATMENT
UNC/BIX INHIBITORY TREATMENT
p53 INHIBITORY TREATMENT
IFN-g/CD8
TNF-a/CD8
IL-2/CD8
0.15% 0.04% 0.08% 0.71% 0.64% 0.22% 3.30% 5.03% 0.13% 1.01% 1.51% 1.18%
0.34% 0.41% 0.12% 1.58% 4.03% 0.37% 1.84% 2.90% 0.51% 1.04% 2.03% 0.45%
0.78% 0.15% 0.01% 2.67% 2.95% 0.23% 5.04% 1.85% 0.98% 3.12% 2.11% 0.03%
RESOLVED PATIENTS T2
2.50% 2.80% 0.33% 4.18% 4.50% 0.62% 9.40% 4.70% 1.54% 5.15% 3.92% 0.53%
6.37% 3.61% 0.29% 11.47% 8.27% 0.72% 15.34% 5.78% 0.55% 8.71% 2.12% 0.85%
0.72% 0.95% 0.10% 0.74% 2.57% 0.03% 1.08% 1.99% 0.05% 1.01% 2.58% 0.08%
CHRONICALLY-EVOLVING ACUTE PATIENTS (T1)
5.81% 3.65% 1.05% 4.76% 3.11% 0.78% 3.12% 1.74% 1.35% 12.80% 10.95% 4.95%
1.50% 0.92% 0.19% 2.41% 0.98% 0.23% 4.07% 0.91% 0.18% 4.65% 2.45% 0.42%
0.72% 0.75% 0.46% 1.68% 1.17% 0.88% 1.90% 0.73% 0.23% 1.22% 1.67% 0.55%
DAA-TREATED CHRONIC PATIENTS (EOT)
0.60% 1.23% 0.03% 1.23% 2.79% 0.11% 1.16% 1.94% 0.03% 1.07% 2.73% 0.10%
0.58% 0.48% 0.42% 0.80% 0.81% 0.37% 1.05% 1.03% 0.23% 1.15% 0.98% 0.48%
0.26% 0.22% 0.06% 0.40% 0.59% 0.09% 0.53% 0.73% 0.23% 0.31% 0.46% 0.09%

Supplementary Fig. 11. Representative plots of cytokine production in the presence or absence of the HMTs/p53 inhibitors.

Cytokine production by virus-specific CD8+ T cells was measured after 10-days culture with HCV-NS3 peptides, in the presence or absence of the EZH2 (Enhancer of Zeste 2) inhibitors GSK126 (GSK) and EPZ005687 (EPZ), of the Euchromatic histone-lysine N-methyltransferase 2 (EHMT2/ G9a) inhibitors UNC0638 (UNC) and BIX01294 (BIX) and in the presence of the p53 inhibitor pifithrin-alfa. The figure illustrates 3 patients of each category, namely T2/late chronics, T2/late resolvers, T1/early chronically evolving acute patients and T2/late resolved patients after DAA treatment. Each horizontal sequence of plots illustrate results derived from an individual patient. To allow better visualization of the results, especially in patients with low levels of response, positive dots delimited by inner squares were enlarged by using the DIVA 7.0 software, applying the same level of magnification to all plots in order to maintain comparability among different patients. Top panels show the gating strategy used for flow cytometry data also in Figures 5e, 5f, 7d-7l, 8b, 8c and Supplementary Figures 4e, 5, 6e, 7a, 7b, 13a and 13b.

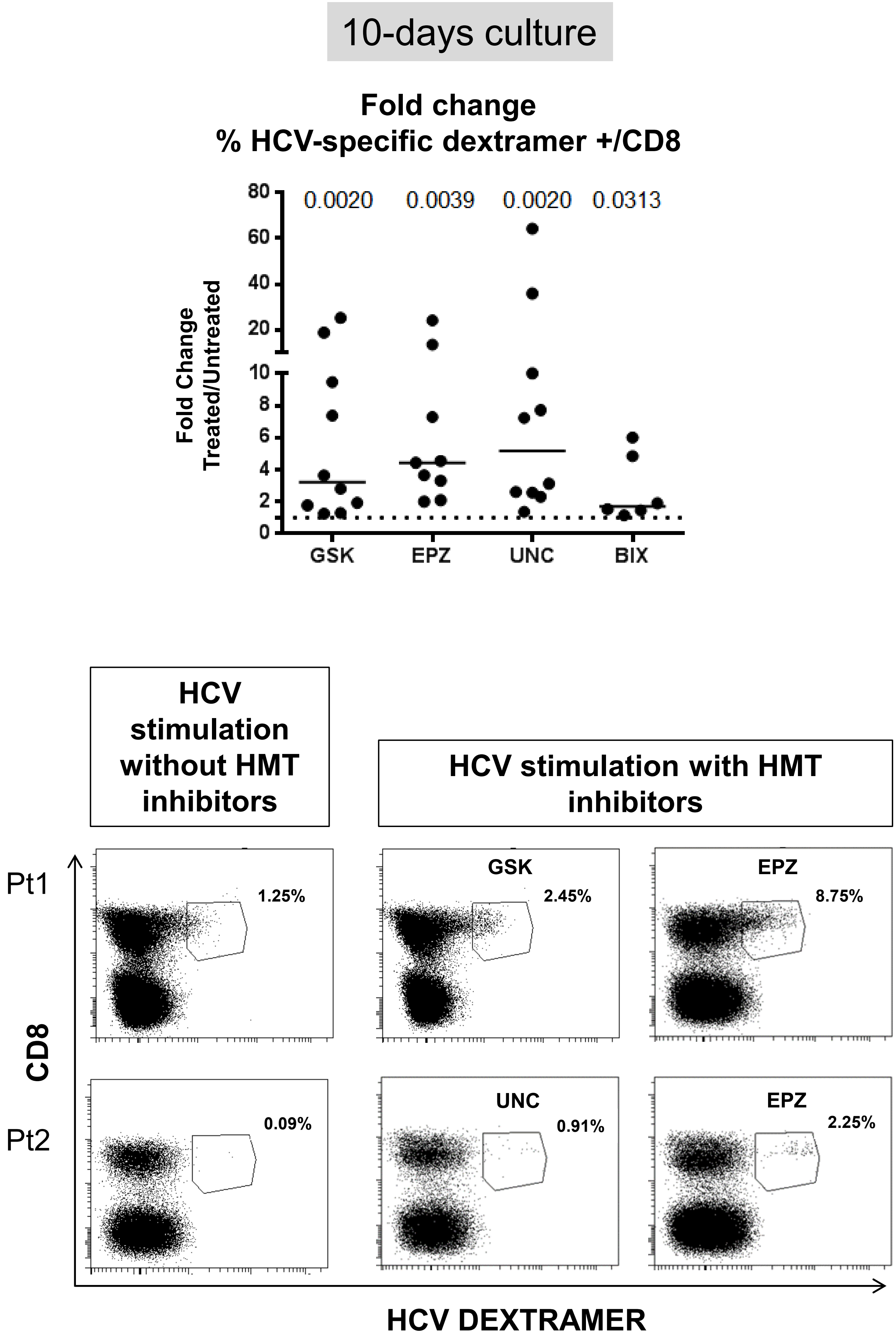

Supplementary Fig. 12. Impact of HMTs inhibitors on the proliferative potential of late exhausted HCV CD8+ T cells.

PBMC from chronic HCV patients (T2/late) were stimulated with HLA-A2-restricted HCV peptides in the presence or absence of HMT inhibitors for 10 days. T cells were stained with the corresponding HLA class-I dextramers prior to flow-cytometric analysis. The plot shows the ratio (fold change) between HMT inhibitor treated vs untreated HCV-specific CD8+ T cells from individual patients (shown as single dots). Horizontal lines represent median values. Statistics by the Wilcoxon signed-rank test. In the bottom plots, examples of dextramer-stained HCV-specific CD8+ T cells untreated or treated with the indicated HMT inhibitors after 10 days of culture with HLA2 restricted HCV-specific peptides.

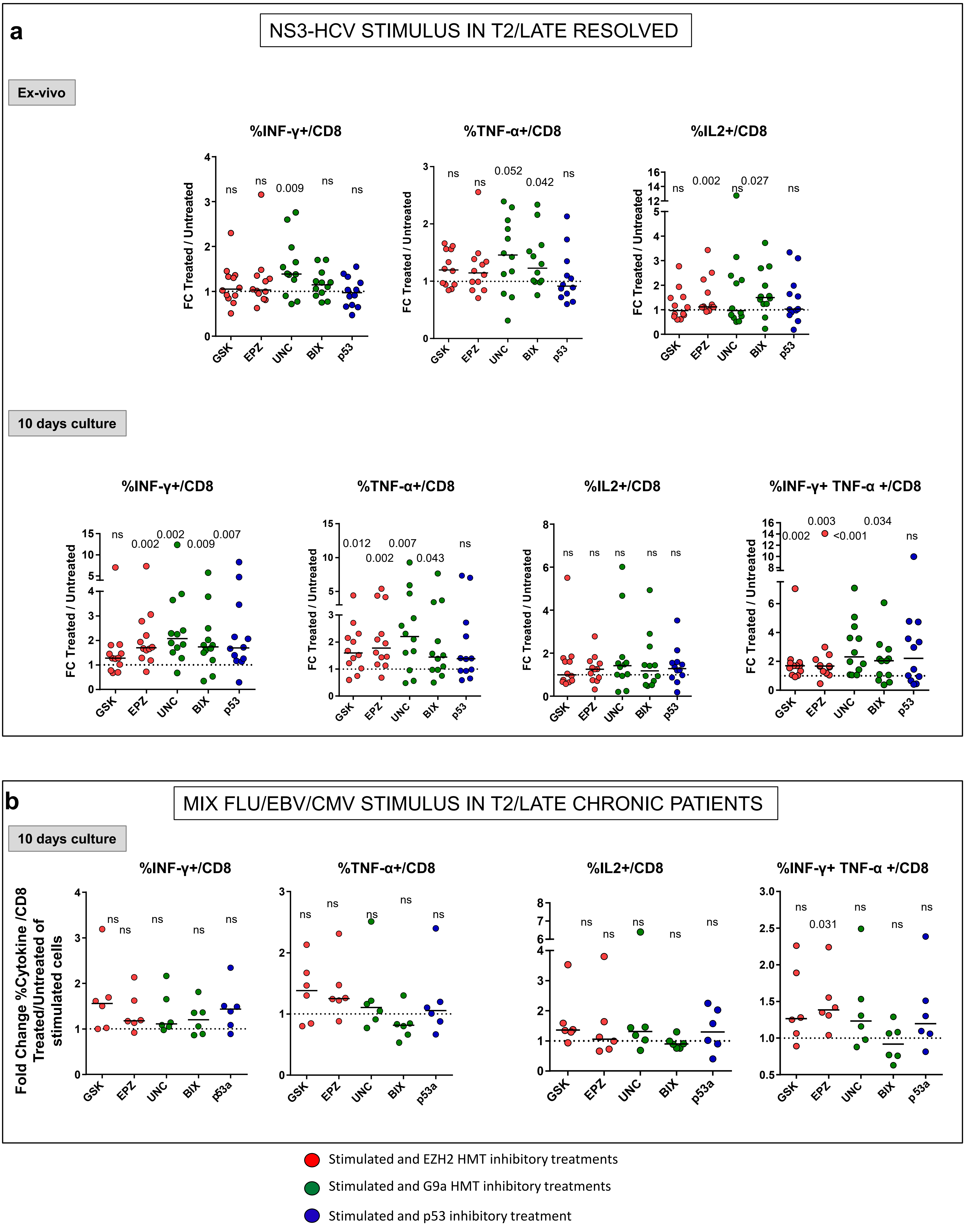

Supplementary Fig. 13. Effect of HMTs/p53 inhibitors on cytokine production in late resolved and chronic patients. **a**, PBMCs from resolved (T2/late) HCV patients (n=12) were stimulated for 40h and for 10 days with HCV-NS3 peptides in the presence or absence of the EZH2 inhibitors GSK126 (GSK) and EPZ005687 (EPZ), *red dots*, of the G9a inhibitors UNC0638 (UNC) and BIX01294 (BIX), *green dots*, and of the p53 inhibitor pifithrin-alfa (p53), *blue dots*. This was followed by co-staining for IFN-γ, IL2 and TNFα to analyze the anti-viral T cell function. **b**, PBMCs from chronic (T2/late) HCV patients (n=6) were stimulated for 10 days with FLU/EBV/CMV peptides cultured as in **a**. Data are presented as ratio between cytokine positive CD8+ T cells detected in treated *vs.* untreated cultures and displayed as fold change in individual patients (shown as single dots). Horizontal lines represent median values. Statistics by the Wilcoxon signed-rank test.

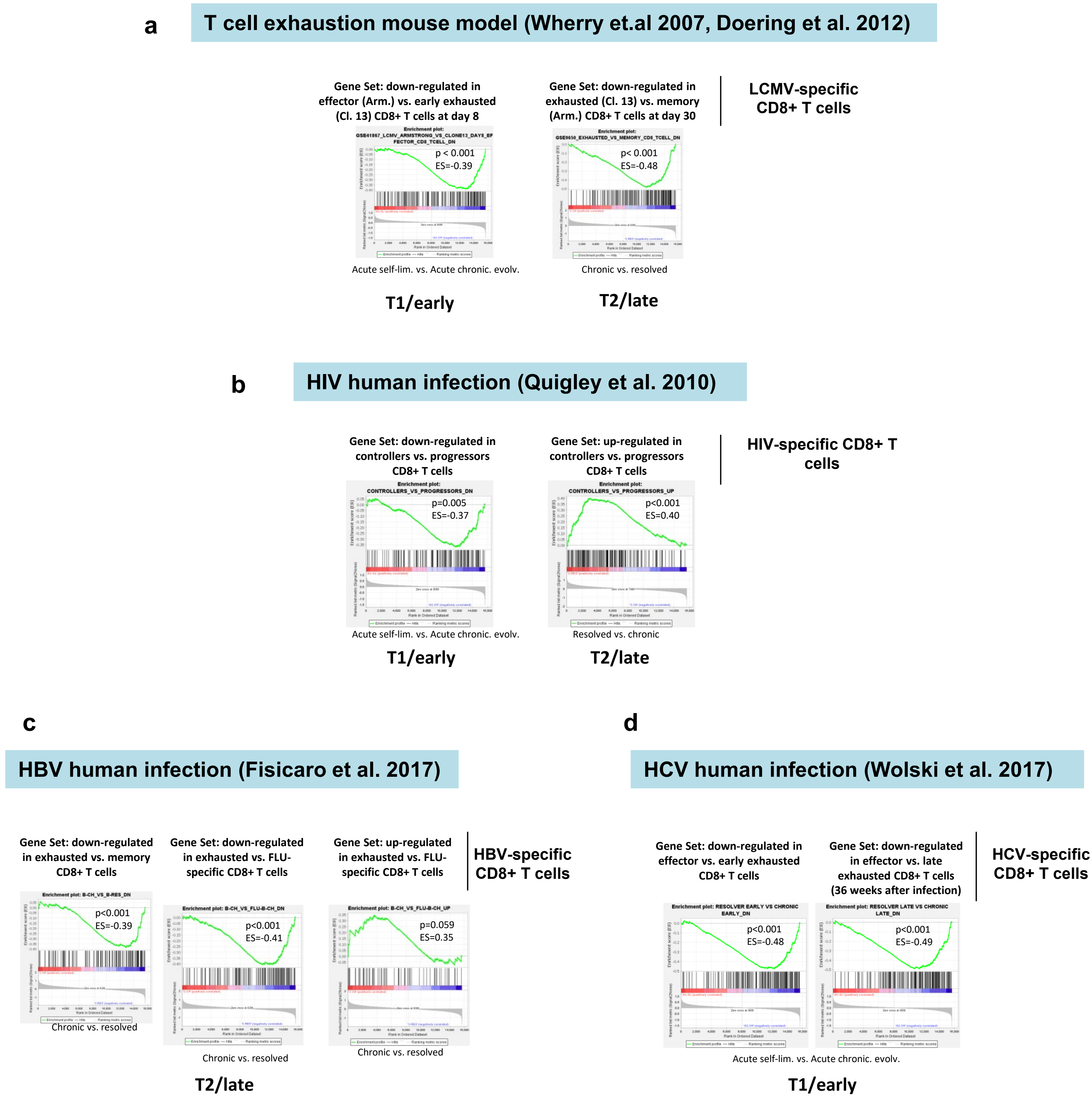

Supplementary Fig. 14. Comparison of HCV specific CD8+ T cells gene expression profiles with different mouse and human models of T cell exhaustion.

Significant GSEA enrichment plots of virus-specific CD8+ T cells transcriptome profiles from chronically-evolving *vs.* self-limited (T1/early) or chronic *vs.* resolved (T2/late) HCV patients, as compared with published gene signatures from:

a. early exhausted *vs.* functional effector virus-specific CD8 T cells at day 8 of LCMV infection, and exhausted *vs.* memory CD8 T cells at day 30 of infection (Wherry et al. 2007 and Doering et al., 2012); **b**. virus-specific CD8 cells from HIV controllers vs HIV progressors (Quigley et al., 2010); **c.** exhausted vs memory HBV-specific CD8 cells from chronic vs resolved HBV infections and vs Flu-specific CD8 cells from healthy subjects (Fisicaro et al. 2017); **d.** HCV-specific CD8 T cells from early HCV infections with chronic *vs.* self-limited evolution (Wolski et al. 2017).

Supplementary Fig. 15

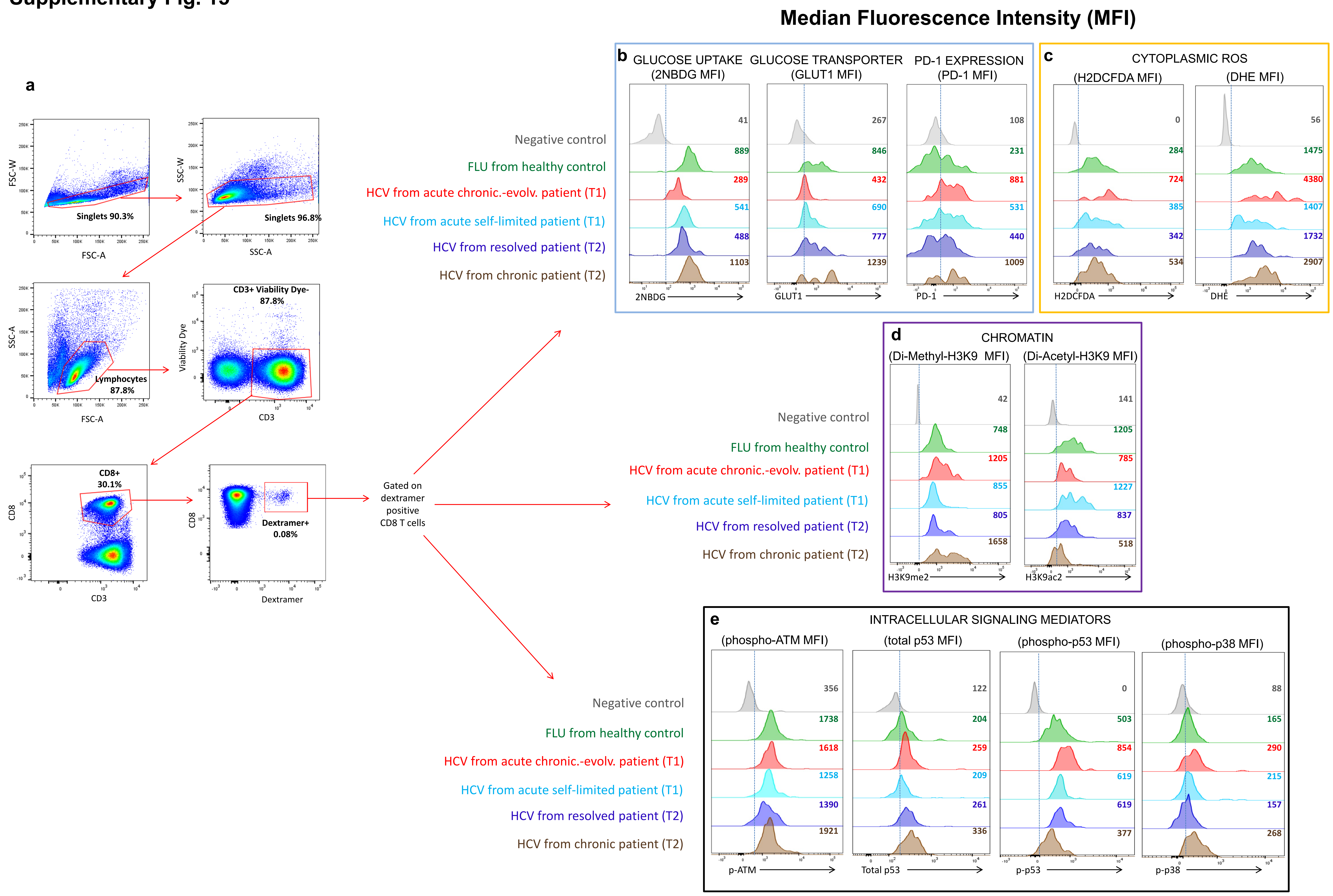

Supplementary Fig. 15. MFI assessment strategy for dextramer positive CD8+ T cell analyses.

a. Left panels show the gating strategy used for flow cytometry analysis of dextramer positive cells also in Figures 2b, 2c, 3a-3c, 4b-4e, 6c, 6e, 8a and Supplementary Figures 4d, 9a-9c, 10a-10f and 12. The same gating strategy was used for flow cytometry analysis of total CD8 positive cells also in Figures 5a-5d, 7a-7c, 7m and Supplementary Figures 6a-6d.

b. The panel illustrates examples of histogram plots of dextramer positive cells showing glucose import/GLUT-1/PD-1 median fluorescence intensity after PBMC overnight stimulation with anti-CD3/anti-CD28 from all patient categories (acute with T1/early chronic evolution, in *red*, acute with T1/early self-limited evolution, in *light blue*, spontaneously T2/late resolved, in *blue* and T2/late chronically infected, in *brown*). *Grey* histograms represent negative controls of FLU specific CD8+ T cells (*green*) from a healthy donor, for all measured parameters obtained as fluorescence minus one (FMO) (see “Flow cytometry measurement controls” in the Methods section**)**. **c.** Cytoplasmic ROS detected with the H2O2 sensitive dye H2DCFDA and the anion superoxide sensitive probe DHE analyzed as in **b**. **d.** Di-methyl-H3 and di-acetyl-H3 expression investigated as in **b** and after 10 days stimulation with HCV or FLU peptides, respectively. **e.** Intracellular signaling transducer expression (phosphorylated ATM, total p53, phosphorylated p53 and phosphorylated p38 MAPK) examined as in **b**.
