# Supplementary material for: Targeting p53 and histone methyltransferases restores exhausted CD8+ T cells in HCV infection
Source: Nat Commun. 2020 Jan 30;11:604. doi: 10.1038/s41467-019-14137-7 (PMC6992697; doi:10.1038/s41467-019-14137-7)
Supplement: Supplementary file 3 — Description of Additional Supplementary Files [file 41467_2019_14137_MOESM3_ESM.docx]

**Description of Additional Supplementary Files**

File Name: Supplementary Data 1

Description: Functional analysis results from Topological pathway analysis/ T1 Comparison (Acute chronically-evolving vs. acute self-limited patients) sheet; T2 Comparison (Chronic vs. resolved patients) sheet; Gene List sheet in T1 Comparison (Acute chronically-evolving vs. acute self-limited patients); Gene List sheet in T2 Comparison (Chronic vs. resolved patients).

File Name: Supplementary Data 2

Description: Functional analysis results from GSEA/ T1 Comparison (FDR for Significance) sheet; T1 Comparison (Biological categories) sheet; T2 Comparison (FDR for Significance) sheet; T2 Comparison (Biological categories) sheet; Gene List sheet in T1 Comparison; Gene List sheet in T2 Comparison.

File Name: Supplementary Data 3

Description: Patient characteristics; DAA-treated patient characteristics.
